# Long-term cure of soft tissue sarcoma with pegylated-liposomal doxorubicin after doxorubicin and ifosfamide failure

**DOI:** 10.1186/s13569-018-0111-0

**Published:** 2019-01-15

**Authors:** Malvi Savani, Paari Murugan, Keith M. Skubitz

**Affiliations:** 1 Department of Medicine, University of Minnesota Medical School, Office Mayo Mail Code 480, 420 Delaware St. SE, Minneapolis, MN 55455 USA; 2 Masonic Cancer Center, Minneapolis, MN USA; 3 Department of Laboratory Medicine and Pathology, University of Minnesota Medical School, Minneapolis, MN USA

**Keywords:** Pegylated-liposomal doxorubicin, Doxorubicin, Sarcoma

## Abstract

**Background:**

Doxorubicin is one of the most active drugs available for the treatment of sarcoma. Pegylated-liposomal doxorubicin (PLD) is a formulation of doxorubicin in which the doxorubicin is encapsulated in liposomes coated with methoxypoly (ethylene glycol); this formulation results in decreased uptake by the reticuloendothelial system, higher concentrations of drug in tumor, and less toxicity, including reduced cardiotoxicity, nausea, alopecia, and myelosuppression. No premedication is necessary. While PLD has a better toxicity profile than free doxorubicin, there is no consensus on the relative efficacy of PLD and free doxorubicin in sarcoma.

**Case presentation:**

In this report, we describe a patient with high-grade metastatic soft tissue sarcoma with rapid recurrence after adjuvant treatment with free doxorubicin, cisplatin, ifosfamide, and dacarbazine. Second-line treatment with PLD resulted in long-term disease remission during a 20-year follow-up period. Mucositis and hand-foot syndrome were controlled by adjustment of dose and treatment interval.

**Conclusions:**

This case illustrates the curative potential of PLD after failure of free doxorubicin and the absence of long term cardiotoxicity with PLD. As with all drugs, individual adjustment of dose and treatment interval is important.

## Background

The median survival of patients with metastatic soft tissue sarcomas (STS) is only 12–15 months [1]. Doxorubicin is one of the most active drugs available for treatment of STS [2–6], although cardiotoxicity can be dose-limiting. Pegylated-liposomal doxorubicin (PLD) is a formulation of doxorubicin in which the doxorubicin is encapsulated in liposomes coated with methoxypoly (ethylene glycol). Unlike doxorubicin, PLD’s uptake by the reticuloendothelial system is decreased, resulting in different pharmacologic properties, including a longer half-life in blood and different toxicities [7–10]. PLD is associated with less cardiotoxicity, nausea, alopecia, and myelosuppression than free doxorubicin; this reduced toxicity obviates the need for premedication [6, 9–14]. PLD’s main toxicities are hand-foot syndrome, low risk of infusion reaction, and some fatigue [6, 9–11, 13, 14]. It has been hypothesized that the symptoms of the infusion reaction, which are associated with transient neutropenia, reflect neutrophil sludging in the microvasculature as observed with hemodialysis neutropenia [15]. We do not routinely pre-medicate patients receiving PLD. In addition, PLD has been shown to localize to implanted tumors in animals [16] and deliver more doxorubicin to the tumor than free doxorubicin in Kaposi sarcoma, prostate cancer, and breast cancer [9, 17, 18]. While PLD has a better toxicity profile than free doxorubicin, there is no consensus on the relative efficacy of PLD and free doxorubicin in STS because of the small number of trials directly comparing these two agents.

In this report, we describe a patient with high-grade metastatic STS with rapid recurrence after adjuvant treatment with free doxorubicin, cisplatin, ifosfamide, and dacarbazine. The patient’s treatment was changed to PLD, and no recurrence was observed during a follow-up period of more than 20 years. This case illustrates the curative potential of PLD after treatment failure with free doxorubicin and the absence of long-term cardiotoxicity with PLD.

## Case presentation

**Fig. 1:**
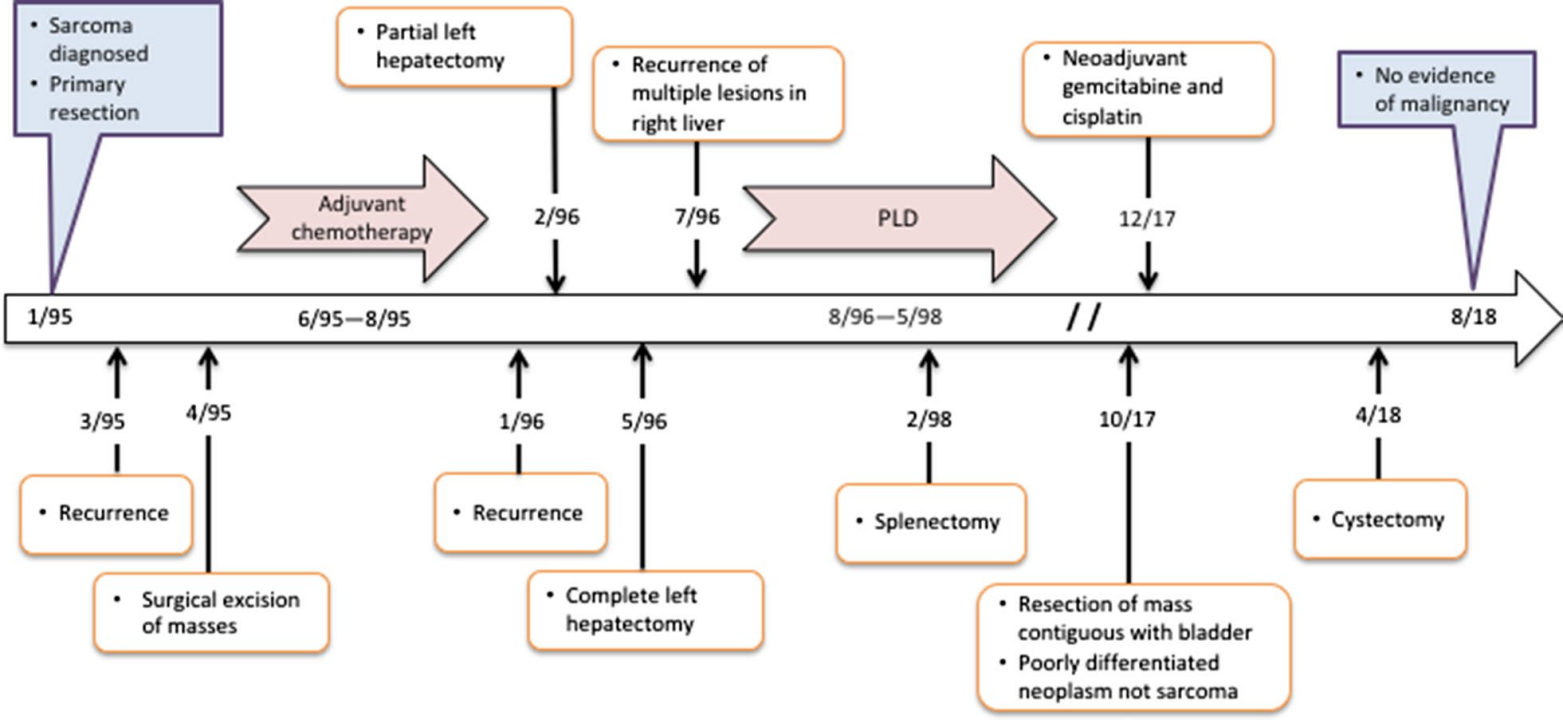
Timeline of treatment

A 37-year-old white man with a past medical history of mitral valve prolapse and gastritis presented with abdominal pain. A computed tomography (CT) scan revealed an 18 cm × 17 cm × 11 cm colonic flexure mass. The patient underwent a resection of the intraabdominal mass with partial small bowel resection, resection of distal transverse and descending colon with enteroenterostomy, as well as colocolostomy, appendectomy and gastrostomy (Fig. 1). Pathology was thought to be consistent with leiomyosarcoma, grade 3/3.

**Fig. 2:**
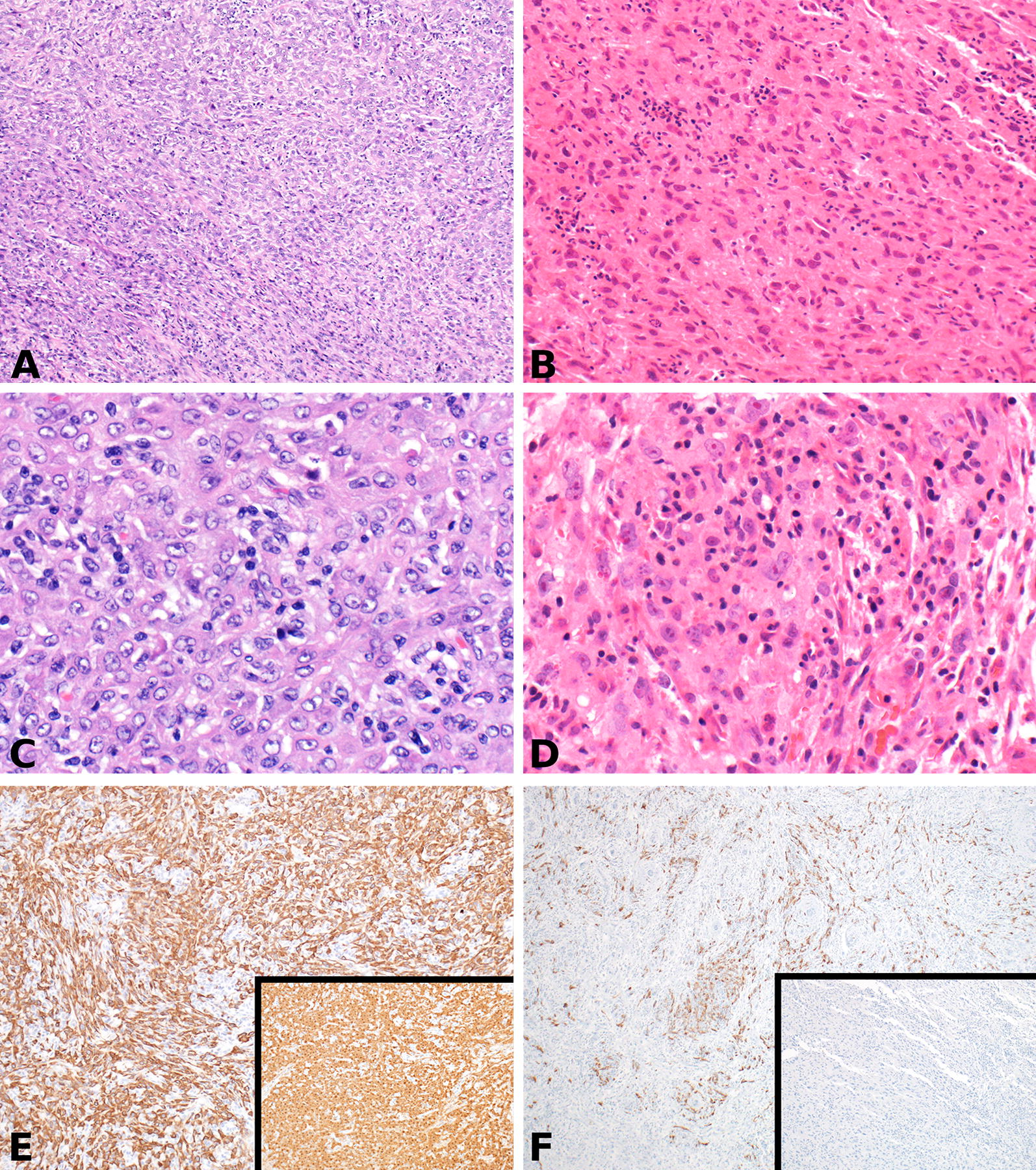
**A**, **C**, and **E** (bladder wall tumor): A highly cellular epithelioid (top right) and spindle cell (bottom left) malignancy involving the outer wall of the urinary bladder (**A**) (H&E ×100). The cells demonstrate round to oval nuclei with variably prominent nucleoli, moderate eosinophilic cytoplasm and indistinct borders (**C**) (H&E ×400). Immunohistochemistry shows diffuse reactivity for cytokeratin AE1/AE3 and calretinin (inset) (**E**) (IHC ×100). **B**, **D**, and **F** (remote abdominal tumor): A moderately cellular malignancy with intermixed spindled and epithelioid cells involving the outer gastric wall (**B**) (H&E ×200). The cells demonstrate irregular nuclei with prominent multiple eosinophilic nucleoli, abundant eosinophilic cytoplasm and indistinct borders (**D**) (H&E ×400). Immunohistochemistry shows reactivity for cytokeratin AE1/AE3 and negative calretinin (inset) (**F**) (IHC ×100)

The gastric wall tumor showed a high-grade spindle cell neoplasm with focal epithelioid features (Fig. 2B). Numerous atypical mitotic figures were noted. The background showed moderate amounts of chronic inflammatory infiltrate. The tumor was originally thought to represent a gastrointestinal leiomyosarcoma. Subsequent studies performed 20 years later (Fig. 2) included immunohistochemical stains showing patchy reactivity for vimentin, cytokeratin AE1/AE3 and cytokeratin 7, while other markers tested, including gastrointestinal stromal tumor and smooth muscle markers were negative. Notably, calretinin was also negative. This histology and immunoprofile were thought to represent an undifferentiated pleomorphic sarcoma (UPS).

Two months after resection of the intra-abdominal mass, the tumor recurred (Fig. 1). He underwent resection of multiple masses in the falciform ligament, left pelvic side wall, small bowel, mesentery, and retroperitoneum. Multiple lymph nodes were also resected. Pathological examination was again thought to be consistent with leiomyosarcoma.

After recovery from surgery the patient received three courses of adjuvant chemotherapy with cisplatin, ifosfamide, dacarbazine, and doxorubicin. This involved 50 mg/m^2^ of cisplatin on day 1; doxorubicin 65 mg/m^2^ on day 1; dacarbazine 300 mg/m^2^ on days 1, 2 and 3; and ifosfamide 2.5 grams/m^2^ a day by continuous infusion for 3 days. This treatment was well tolerated, aside from neutropenic fevers requiring hospitalization.

Three months later, however, a CT scan revealed a 4-cm metastatic lesion in the left lobe of the liver. The patient underwent a partial left hepatectomy and cholecystectomy.

Three months later, a CT revealed multiple 2–3 cm lesions along the margin of the previous partial left hepatectomy. At the time, the patient noted night sweats and low-grade fevers of 99–100 °F, but good appetite and no significant weight loss. The tumor doubled in size during the next 5 weeks and imaging revealed multiple lesions throughout the remaining left lobe. He subsequently underwent a complete left hepatectomy and excision of perigastric lymph nodes. Pathological examination was thought to confirm metastatic grade 3 leiomyosarcoma of the liver. No extrahepatic involvement or lesions in the right lobe were noted by intraoperative ultrasound.

Five weeks later, the patient noted right upper abdominal fullness and pain, left shoulder pain, and presented to our clinic. Pathology review of the earlier resections at our institution and at another large sarcoma center in the US agreed with the diagnosis of leiomyosarcoma. A CT scan showed multiple metastases in the right lobe of the liver and splenic metastases. Immediately before starting PLD, CT imaging revealed multiple metastases in the remaining left lobe of the liver, the largest being 4 × 4 cm, and one spleen nodule. The left ventricular ejection fraction (EF) was 58% by multi-gated acquisition (MUGA). The patient began treatment with PLD at 55 mg/m^2^ monthly. After 2 cycles of PLD a CT scan showed regression of tumor nodules. Because of mucositis and hand-foot syndrome the dose was reduced 10% for cycles 2–4 and 50% for cycle 5; thereafter, the dose was increased to 30.8 mg/m^2^ monthly starting with cycle 6 and the treatment interval lengthened to every 6 weeks starting with cycle 7. The left ventricular EF was 61% by MUGA before cycle 12. After 14 cycles the EF was 62% by MUGA and a CT scan showed a persistent lesion in the spleen. At this point, after 14 cycles of PLD, CT imaging revealed no visible disease in the liver and the spleen nodule that was unchanged in size but had a lower density. Thus, while a complete response by RECIST criteria was evident in the liver, the stable size of the spleen nodule indicated a partial response to PLD by RECIST. A splenectomy was performed revealing some viable tumor cells in a necrotic nodule. The patient received 3 more cycles of PLD for a total PLD dose of 595 mg/m^2^ and a total dose of free doxorubicin of 196 mg/m^2^. CT imaging revealed no tumor and MUGA showed an EF of 70%. The patient was followed with interval imaging with no recurrence.

Twenty-two years later, the patient experienced several months of scrotal pain radiating to his urethra and localized to his right testicle. An ultrasound showed a 2.8 cm × 1.6 cm × 0.8 cm mass on the outer surface of the bladder. Fine needle aspiration of the mass showed poorly differentiated carcinoma that was CK7+/CD20+ and histologically compatible with urothelial origin. A PET-CT revealed an intense hypermetabolic pelvic mass contiguous with the right side of the bladder. An exploratory laparotomy, intraabdominal mass resection, and partial cystectomy were performed.

Pathologic examination of the bladder wall tumor showed a 5.5 cm high-grade malignancy with epithelioid and sarcomatoid features (Fig. 2A). Margins were negative on the perivesical soft tissue mass, and 16 of 16 lymph nodes were negative for malignancy. Mitotic activity was high, and the background showed moderate chronic inflammation. It appeared predominantly located in the outer half of the bladder wall, involving the muscularis propria and perivesical fat. No mucosal involvement or in situ urothelial carcinoma was identified. The tumor was diffusely and strongly positive for calretinin, vimentin and cytokeratin. It was negative for all other markers tested, including gastrointestinal stromal tumor and smooth muscle markers. Given the presence in the bladder and co-expression of keratin and vimentin, it was possible that this represented a urothelial or a urachal remnant-based carcinoma with sarcomatoid differentiation. However, the location of the tumor (posterior dome, per imaging), lack of associated mucosal lesion, positive calretinin stain, and lack of reactivity for urothelial markers such as GATA3, p63, and CK5/6 were unusual for the above diagnosis. The diffuse calretinin reactivity, in conjunction with co-expression of keratin and vimentin, raised the possibility of mesothelial differentiation/origin. While the morphology was not incompatible with a poorly differentiated biphasic mesothelioma, the overall clinical presentation, lack of serosal continuity and non-reactivity for ancillary mesothelial markers (WT1, D2-40 and CK 5/6) precluded a definitive diagnosis of the same.

The morphology and immunohistochemical staining patterns of the remote gastric lesion were compared to that of the bladder neoplasm. Both were poorly differentiated neoplasms, but the high magnification cytology and calretinin reactivity were dissimilar; there was no clear evidence that the tumor on the bladder wall represented a recurrence of the remote malignancy. His hospital course was complicated by Citrobacter urinary tract infections and intraabdominal abscesses requiring three intraabdominal drains and intravenous antibiotics. EF by ultrasound was normal at 55–60%. He subsequently underwent neoadjuvant gemcitabine and cisplatin followed by total cystectomy; the cystectomy specimen showed no evidence of residual tumor. He continues to do well 4 months after surgery, with no evidence of tumor recurrence.

## Discussion and conclusions

Doxorubicin remains one of the most active agents with therapeutic activity against advanced STS [2–6]. Numerous phase II and III trials have been conducted showing superior response rates using a combination of doxorubicin and other agents in the treatment of metastatic STS, though demonstration of a clear survival benefit has been elusive. A phase III trial conducted by Judson et al. found no statistically significant improvement in overall survival with the addition of ifosfamide to doxorubicin for palliative treatment of advanced STS [19]. However, this study did show a higher response rate (26% vs 14%) and longer median progression-free survival (7.4 months vs 4.6 months) in the combination cohort, raising the argument in favor of adding ifosfamide to doxorubicin [2, 19]. Our case failed adjuvant treatment with a combination of doxorubicin, cisplatin, ifosfamide, and dacarbazine, but was cured by subsequent treatment with PLD and resection of one remaining nodule. One could question exactly what it means to be “doxorubicin resistant/refractory.” Technically, we did not demonstrate tumor growth while the patient was receiving doxorubicin. However, the patient was treated with 3 cycles of a doxorubicin containing regimen starting at a time when there was no tumor detectable by CT imaging. Three months after stopping the doxorubicin containing regimen, CT imaging revealed a sizable tumor burden. One must conclude that either the tumor was not inhibited by the doxorubicin containing regimen or that it kept residual tumor cells dormant until it was stopped and then those tumor cells grew very rapidly, or alternatively, some tumor cells were killed by the doxorubicin containing regimen, but the remaining cells grew very rapidly over 3 months. In any of these cases, it seems very unlikely that it would have been possible to cure the patient with further doxorubicin if 3 cycles starting with no residual tumor detectable did not, especially given the size of the tumor burden and the issue of dose related cardiotoxicity.

PLD is a unique formulation of doxorubicin in that the agent is contained in liposomes coated with hydrophilic methoxypoly (ethylene glycol), that diminishes uptake of the agent by the reticuloendothelial system and consequently increases the half-life of the drug in blood to approximately 50–60 h [7, 9, 20]. PLD localizes to tumors due to increased vascular permeability, resulting in greater drug concentration in tumor in comparison to free doxorubicin [9, 16–18]. A number of trials have demonstrated activity of PLD in a variety of tumors, including sarcomas (Table 1) [6, 10, 13, 14, 21, 22]. A phase II trial by the EORTC Soft Tissue and Bone Sarcoma Group compared the results of 50 patients treated with PLD at 50 mg/m^2^ every 4 weeks and 44 patients treated with doxorubicin at 75 mg/m^2^ every 3 weeks for advanced STS; they found that PLD had equivalent activity as doxorubicin with an improved toxicity profile, including lower incidence of myelosuppression and alopecia. However, a higher incidence of palmar-plantar erythrodysesthesia was noted in the cohort receiving PLD [6]. This study had a high proportion of gastrointestinal stromal tumors. They also concluded that further studies of PLD in combination with other drugs should be considered.

**Table 1:** Summary of clinical trial results of PLD in sarcoma

Study	Disease	Dosing regimen	Organizer/sponsor	# of patients	Responses and toxicities	References
Phase 2	STS (many patients had poor prognostic features, including low-grade tumors	PLD 50 mg/m^2^ every 4 weeks		13	None Treatment responses possibly affected by poor prognostic features	Garcia et al. [13]
Phase 2	Advanced and/or metastatic STS Patients were previously treated with an anthracycline-based chemotherapy	PLD 30–50 mg/m^2^ every 3 weeks		25	3 PRs, 4 minor responses, and 17 patients with SD	Toma et al. [22]
Phase 2 randomized	Advanced STS, with a high proportion of gastrointestinal stromal tumors	PLD 50 mg/m^2^ every 4 weeks Doxorubicin 75 mg/m^2^ every 3 weeks	EORTC Soft Tissue and Bone Sarcoma Group	94 (50 PLD, 44 doxorubicin)	PLD had equivalent activity as doxorubicin with an improved toxicity profile, including lower incidence of myelosuppression and alopecia. However, a higher incidence of palmar-plantar erythrodysesthesia was noted in the cohort receiving PLD	Judson et al. [6]
Phase 2	Previously treated sarcomas or sarcomas considered unresponsive to chemotherapy	PLD 55 mg/m^2^ with subsequent dose adjustment		47	3 CR or PR and 15 clinical benefit Treatment was generally well tolerated, and mucositis and hand-foot syndrome were the dose-limiting toxicities	Skubitz [10]
Phase 2	Advanced leiomyosarcoma of the uterus	PLD 50 mg/m^2^ every 4 weeks		31	CR in 1, PR in 4, and SE in 10 patients	Sutton et al. [21]
Retrospective analysis	Metastatic STS	Initial PLD 40–60 mg/m^2^ every 4 weeks		11	PR in 6 with extended time to progression, SD in 2, and PD in 3 (One patient was progression free for 60 months after receiving seven cycles of PLD)	Grenader et al. [14]

In addition to its activity in STS, PLD has an improved toxicity profile as compared with free doxorubicin. An important limiting toxicity of doxorubicin is cardiotoxicity. In contrast, PLD has much less cardiotoxicity [10, 12, 20, 23–25]. A recent retrospective study [12] showed no definitive doxorubicin-induced clinical heart failure (HF) in 56 patients receiving a cumulative dose of free doxorubicin and PLD comparable or higher than the dose our case received (PLD: 595 mg/m^2^ and free doxorubicin: 196 mg/m^2^). In this retrospective study, 56 patients received a cumulative dose of free doxorubicin and PLD of > 450 mg/m^2^, 49 patients received > 500 mg/m^2^, 14 > 1000 mg/m^2^ and 5 > 1400 mg/m^2^. While modest changes in EF were noted over time in the absence of clinical signs or symptoms of HF, EF was not considered a useful predictor of doxorubicin-induced cardiotoxicity, at least in the case of PLD [12]. Our case’s EF remained within or above the normal range before, during, and after PLD treatment for the 20-year follow-up period. While dexrazoxane is occasionally employed during initiation of bolus doxorubicin to reduce long-term cardiotoxicity, this is not required with administration of PLD, eliminating any concern that dexrazoxane might decrease anti-tumor activity.

PLD is a different formulation of doxorubicin with different pharmacologic properties and different toxicities than bolus free doxorubicin. In addition to reduced cardiotoxicity, PLD has markedly reduced nausea, alopecia, and myelosuppression, and no anti-emetics or other pre-medications or growth factors are usually needed. A small number of patients experience an infusion reaction in the first few minutes of the first treatment, that manifests as shortness of breath or low back pain. It has been suggested that the symptoms of the infusion reaction, which are associated with transient neutropenia, reflect neutrophil sludging in the microvasculature as observed with hemodialysis neutropenia [15]. Pre-medications have not been shown to prevent this reaction, which usually only occurs with the first treatment. The main toxicities of PLD are mucositis, hand-foot syndrome, and mild fatigue. Notably, not all liposomal formulations are the same; non-pegylated liposomal anthracyclines have different pharmacologic properties and lack some of the favorable properties of PLD.

While some early studies used doses of PLD as high as 55 mg/m^2^, this dose is usually too high for a monthly treatment schedule; a more typical monthly starting dose is 45 mg/m^2^. When dose-limiting toxicities are seen, the next dose should be delayed until there is no pain from mucositis or hand-foot syndrome. Cardiotoxicity is rare, and there is no study demonstrating that monitoring the EF as a predictor of cardiotoxicity is useful [10, 12, 24, 25]. In conclusion, PLD has a more favorable toxicity profile, including reduced incidences of cardiotoxicity, nausea, myelosuppression, and alopecia. As a result no pre-medications or growth factor is required [6–15, 20, 24, 25]. Furthermore, PLD results in higher concentrations of drug in tumor than free doxorubicin [9, 16–18]. Our case highlights that PLD can cure a patient with a doxorubicin-resistant tumor, demonstrating that in some cases PLD is more efficacious than free doxorubicin with a more favorable toxicity profile. As with all drugs, individual adjustment of dose and treatment interval is important.

## Abbreviations

PLD: pegylated-liposomal doxorubicin
STS: soft-tissue sarcoma
EF: ejection fraction
HF: heart failure
